# Additional green light induces shade response symptoms in *Brassica rapa* as evidenced by increased lateral root spread

**DOI:** 10.17912/micropub.biology.000723

**Published:** 2022-12-20

**Authors:** Kaylynn G Imsande, Janet M Batzli

**Affiliations:** 1 Department of Biology Core Curriculum (Biocore), University of Wisconsin – Madison, WI, USA

## Abstract

In some plant species, green light (500 to 570 nm) has been shown to act as a shade signal, which stimulates non-photosynthetic photoreceptors to initiate a response that promotes shading symptoms, including lateral root formation. No studies to date have examined whether green light induces shading symptoms in *Brassica rapa* specifically. Here, we report increased hypocotyl length, root width, and increased width:depth ratio of root architecture in plants grown under additional green light compared to red and blue light, and white light alone. Results indicate that green light acts as a shade signal in *B. rapa* to induce shading symptoms, including wider roots.

**Figure 1.**
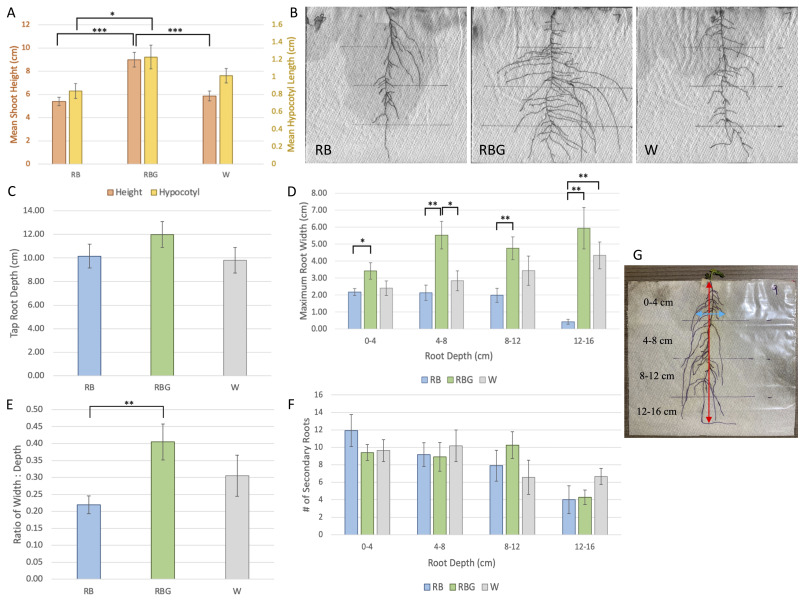
**(A)** Mean shoot and hypocotyl measurements for red and blue (RB; 135 PAR), red, blue and green (RBG; 190 PAR), and white (W; 135 PAR) light treatments (n = 16 plants). There is a significant increase in mean shoot height in RBG plants compared to RB and W plants (p = 2.70e-5 & p = 0.0003, respectively). There is also a significant increase in mean hypocotyl length in RBG plants compared to RB plants (p = 0.022). There is no significant difference in mean shoot height or hypocotyl length between RB and W plants. In RB treatment, 3/16 plants were flowering; in RBG, 14/16; in W, 2/16. **(B)** Scans of roots of an RB, RBG, and W treatment plant. RBG treatment roots grow wider laterally than in RB or W treatments. **(C)** Mean tap root depth for RB, RBG, and W treatments. ANOVA revealed no significant difference in mean tap root depth between any of the treatments. **(D)** Mean maximum root width per quartile of root depth for RB, RBG, and W treatments. ANOVA revealed significant differences (p < 0.05) in mean maximum root width per quartile between treatment groups in all quartiles. Post-hoc analyses then revealed the following: 0-4 cm: significant increase in RBG width compared to RB (p = 0.021); 4-8 cm: significant increase in RBG width compared to RB and W (p = 0.001 & 0.014, respectively); 8-12 cm: significant increase in RBG width compared to RB (p = 0.004); 12-16 cm: significant increase in RBG and W width compared to RB (p = 0.009 & 0.009, respectively). **(E)** Mean ratio of width:depth for RB, RBG, and W treatments. ANOVA and post-hoc analyses revealed a significant increase in ratio in the RBG plants compared to the RB plants (p = 0.004). There is no significant difference between W plants and RB or RBG plants. **(F)** Mean number of secondary roots per quartile for RB, RBG, and W treatments. ANOVA revealed no significant difference in mean number of secondary roots per quartile between treatments in any quartile. **(G)** Example diagram of root quartiles. Red arrow represents tap root depth (measured perpendicular from top of bag); blue arrow represents maximum root width in 0-4 cm quartile. RB = red+blue light; RBG = red+blue+green light; W = white light. In panels C-F: n = 12 for RB, n = 10 for RBG, n = 11 for W. All error bars represent ± 1 SE of the mean. p < 0.05 = *, p < 0.001 = **, p < 0.001 = ***.

## Description

Light, including quantity, quality, direction, and duration of irradiance, is an essential component of plant growth and development, including light-induced adaptive changes, known as photomorphogenesis (Carvalho *et al* . 2011). Quality (wavelength) of light has a significant impact on what specific adaptations occur. Added green wavelengths (495-570 nm) have been found to act as a shade signal to plants, resulting in an adaptive response that enables the plant to survive in a low-light environment (Smith *et al* . 2017; Zhang *et al* . 2011). Supplemental green light paradoxically causes low-light symptoms in *Arabidopsis thaliana* plants even when the addition of green light increases the overall fluence rate of the growth light (Zhang & Folta 2012). Specifically, increasing the green-light ratio in a constant background of blue and red light has been shown to activate a response similar to a phytochrome-mediated FR light-induced shade avoidance syndrome (Smith *et al* . 2017).

Multiple studies have shown that cryptochromes (non-photosynthetic photoreceptors) can sense a decreasing ratio of blue:green wavelengths, which induces these low-light phenotypic adaptations (Franklin & Whitelam 2005; Smith *et al* . 2017; Sellaro *et al* . 2010; Alarcón *et al* . 2019). Examples of developmental adaptations observed in *Arabidopsis thaliana* include enhanced growth of hypocotyl and petioles, more erect position of leaves, and early flowering (Casal 2012; Sellaro *et al* . 2010). While the above-ground low-light response is well documented, the below-ground effects of a low-light response are relatively understudied and poorly understood. Additionally, it is known that green light can act as a shade signal in species such as *Arabidopsis thaliana* , though it is unknown if green light acts as a shade signal to induce a low-light symptoms in *B. rapa* specifically (Zhang *et al* . 2011).

Like *Arabidopsis thaliana* , *Brassica rapa* are sun-adapted, fast-growing plants. Therefore, under low-light conditions, *B. rapa* undergoes etiolation and/or phototropism in search of a light source. Taller plants require a strong anchor to maintain shoot structure and support growth. Therefore, we believe there is enhanced lateral growth of roots under low-light conditions with the functional consequence of supporting taller, etiolated shoots (Zhang *et al* . 2011; Alarcón *et al* . 2019). We thus hypothesized that *B. rapa* grown under a combination of red, blue, and green light would exhibit root architecture that is wider than in *B. rapa* grown under red and blue light, or white light. To test this hypothesis, we grew *B. rapa* seeds under three treatment conditions (red and blue light (RB), red, blue, and green light (RBG), and white light alone (W)) to determine if there were differences in lateral root architecture between treatments. RB and W treatments were maintained at equal fluence rates of 135 PAR, while the RBG treatment was maintained at a higher fluence rate of 190 PAR.

We observed a statistically significant increase in mean maximum root width at all rooting depths in the RBG treatment plants compared to the RB treatment plants. There are a few important implications of these results. First, while low-light responses have been observed in *Arabidopsis thaliana* , a low-light response has not yet been demonstrated in *B. rapa* , to our knowledge (Zhang *et al* . 2011). Plants grown in the RBG box responded with longer hypocotyls and earlier flowering as compared to RB (see panel A), indicating that a low-light response is occurring under the additional green light conditions, and that green light acts as a shade signal for above-ground plant development in *B. rapa* . Additionally, this finding provided evidence that the RBG treatment conditions in our experiment may be sufficient to induce a low-light response associated with root growth, and thus would allow us to appropriately address the hypothesis.

Second, while the above-ground effects of a low-light response have been well studied, there is limited research on the effects of a low-light response in plant roots. Because plants grown in the RBG box (i.e., the plants displaying above-ground symptoms of a low-light response) consistently had wider roots throughout the rooting volume than plants grown in the RB box (see panels B, D & E), results indicate that green light treatment does cause plant roots to grow wider. Our results are a snapshot of an interesting phenomenon; fluence rate response experiments with increasing amounts of light and experiments utilizing varying amounts of green wavelengths relative to red and blue wavelengths are the most certain next steps to verify that it is the light quality, as opposed to the light quantity, that is causing changes in the roots. Future experiments would also need to be conducted to determine the physiological mechanism behind these observed phenotypic differences.

There are a few important ramifications of our results. As previously stated, effects of green light on plants in general are relatively understudied, and many of the published results are contradictory (Smith *et al* . 2017). For this reason, studies such as this one that examine green light effects (particularly in the roots) and mechanisms by which green light influences plant physiology are novel research and are important for our understanding of the effects of the full spectrum of light wavelengths (and varying ratios of wavelengths) on plant growth and development. Additionally, studies have shown that green light increases water-use efficiency and significantly contributes to photosynthetic carbon assimilation and biomass accumulation in the lower canopy where blue and red wavelengths are severely depleted. As such, there is a strong case for adding green light to LED setups in horticultural systems to promote efficient growth (Smith *et al.* 2017). This research provides insight into other developmental responses associated with additional green light, including earlier flowering (and potentially fruiting) and wider root growth, that may be observed if green light were incorporated into horticultural systems.

In conclusion, the goal of this research was to determine how green light influences root growth and development of *B. rapa* . We found that green light does influence how roots grow. Specifically, green light causes wider root growth in *B. rapa* , with potential functional consequences for support of above-ground shoot growth.

## Methods

***Plant Growth Boxes***

Plants were grown in a cardboard box (45 cm tall by 36 cm wide by 25 cm deep) growth system lined with mylar sheeting to reflect light from all angles and help control fluence rate. The top of the box was fitted to accommodate LED lightbulbs. Two dowels were suspended in each box at a height of roughly 12 cm so the growth bags could be clipped to the dowels and rest at approximately a 45° angle within the box. Resting the bags at a 45° angle encouraged roots to grow against the bottom surface of the Ziploc bag, which prevented roots from sewing into the paper towel.

***Light Treatments***

There were three light treatment groups in this experiment: red + blue light (RB), red + blue + green light (RBG), and white light alone (W). The peak wavelengths were 625 nm, 453 nm, and 522 nm for the red, blue and green LED bulbs, respectively; the white bulb had peaks at 458 nm, 545 nm, 587 nm, and 614 nm (measurements were taken using a Gigahertz Optik MSC15 Spectroradiometer). Each treatment had an individual grow box. The red + blue box was the control (a negative control lacking green light) and the comparison for the RBG treatment box. RB treatment contained 1 red and 1 blue LED lightbulb and was maintained at a fluence rate of 65 PAR (PAR = μmol photons/m ^2^ /s) of red light and 85 PAR of blue light (for a total fluence rate of 135 PAR). Red and blue light, as opposed to white light, was used as the comparison for additional green light because our objective was to carefully control the ratio of blue:green wavelengths present, as literature suggests it is this ratio that is important to induce phenotypic adaptations (Zhang *et al* . 2011). The red + blue + green box was the experimental treatment and contained 1 red, 1 blue, and 2 green LED lightbulbs. This box was maintained at a fluence rate of 60 PAR of red light, 85 PAR of blue light, and 80 PAR of green light (for a total fluence rate of 190 PAR). Blue:green ratio was maintained at approximately 1:1, shown by Zhang *et al* . (2011) to be a sufficient ratio to induce a green light shading response even with a high fluence rate. The white light box comparator included 1 white LED lightbulb and was maintained at a fluence rate of 135 PAR. This box was a methodological control used to verify that there were no unexpected effects occurring in the RB plants and that growth conditions were adequate under RB, thus allowing us to use RB as a comparison for RBG. To carefully control fluence rate, the PAR of each box was checked daily using a portable LiCor light meter (LI-250A).

***Root Growth Bags***

Seeds were germinated in quart-sized Ziploc bags (approximately 16 cm x 16 cm) lined with white paper towel. This technique allowed plants to grow in a system where the roots support the weight of the plant (i.e., not hydroponics), since the reasoning behind enhanced lateral root growth is to support etiolated and/or phototropic shoots (Alarcón *et al* . 2019), while also allowing us to observe roots without disturbing the developing architecture. Grow bags consisted of paper towels cut to fit inside a Ziploc bag with the zipper removed. Hoagland solution was pipetted onto paper towel until saturated (Hoagland & Snyder 1933). A single *B. rapa* seed (Wisconsin Fast Plants Strain 1-033 Standard, Rapid-cycling *Brassica* collection, University of Wisconsin-Madison, Department of Plant Pathology, Madison, WI, USA)) was then placed into the center of the open top of the bag. The seed was covered with a tiny (roughly 1 cm x 1 cm) square of paper towel dampened with Hoagland solution. The purpose of this was to keep the seed moist, thus encouraging germination.

Twelve bags were planted for each treatment box (36 bags total). In the boxes, each bag was clipped to one of the dowels suspended in the box so that the bag rested at a 45° angle. Once all bags were clipped to the dowels, we covered the bags in aluminum foil leaving only the top of the bag and germinating seed open to air and exposed to the LED light(s) above, while preventing new roots from receiving any light and preventing algae and bacteria growth in the bag. Throughout the growth period, Hoagland solution was added as necessary. After 10 days of growth, the experiment was terminated and data was collected (Cleland 1995; Zhang *et al* . 2012).

***Above-Ground Growth Trays***

To verify a low-light response was induced in the *B. rapa* plants under our treatment conditions, we ran an experiment to observe shoot growth since the effects of a low-light response on shoot growth are much better understood. Plants were grown in a soil-less 3:1 mixture of vermiculite and peat moss that allowed plants to grow as they would in normal soil. 16 *B. rapa* seeds were planted in a 4x4 propagation tray filled with vermiculite mixture. Seeds were watered with 0.5 g/L Jack’s Water-Soluble All-Purpose Plant Food nutrient solution until mixture was saturated. The propagation tray was then placed on a small plastic tub covered with felt strips and filled with nutrient solution that served as an automatic watering system. Three trays of 16 seeds were planted and one tray was placed in each treatment box.

Throughout the growth period, plants were checked on and rotated daily to control fluence rate and prevent phototropism. Watering systems were filled with nutrient solution as necessary. After 12 days of growth, plants were harvested and data was collected.

***Data Collection***

To collect data on shoots, we measured hypocotyl length and total height of each plant. We then made observations on whether each plant was flowering. To collect data on roots, growth bags were placed on a backlit surface to trace root architecture onto the bag. Bags were then divided into 4 equal depth quartiles: 0-4 cm, 4-8 cm, 8-12 cm, and 12-16 cm depth. We then measured the deepest point of the main tap root (see panel G). In each quartile, we counted the number of secondary roots branching off the primary tap root and measured the width of the point at which the leftmost and rightmost roots were spread widest in that quartile (see panel G). All data collection was one-way blinded.

***Statistical Analysis***

For each root system, we calculated mean maximum root width of the top two quartiles (0 to 8 cm) and divided this number by the tap root depth to obtain a width to depth ratio of the root system. ANOVA tests were conducted in RStudio to compare means of each dependent variable to determine if differences between the three light treatment groups were statistically significant at p<0.05. When statistically significant differences were found using the ANOVA test, we conducted a post-hoc t-test using the Bonferroni correction to determine where the differences in means arose from.
